# TG221: An Experimental Model for Liver Cancer Prevention and Treatment Approaches

**DOI:** 10.3390/biotech15010009

**Published:** 2026-01-19

**Authors:** Elisa Callegari, Angelo Michilli, Farzaneh Moshiri, Bruno De Siena, Laura Gramantieri, Massimo Negrini, Silvia Sabbioni

**Affiliations:** 1 Department of Translational Medicine, University of Ferrara, 44121 Ferrara, Italy; elisa.callegari@unife.it (E.C.); mshfzn@unife.it (F.M.); ngm@unife.it (M.N.); 2 Department of Life Sciences and Biotechnology, University of Ferrara, 44121 Ferrara, Italy; mchngl@unife.it (A.M.); bruno.desiena@unife.it (B.D.S.); 3 Division of Internal Medicine, Hepatobiliary and Immunoallergic Diseases, IRCCS Azienda Ospedaliero-Universitaria di Bologna, 40138 Bologna, Italy; laura.gramantieri@aosp.bo.it; 4 Laboratorio per le Tecnologie delle Terapie Avanzate (LTTA), Department of Translational Medicine, University of Ferrara, 44121 Ferrara, Italy

**Keywords:** hepatocellular carcinoma, preclinical models, TG221 mouse model, microRNA, miR-221, miRNA therapeutics, sorafenib resistance

## Abstract

Hepatocellular carcinoma (HCC) is a leading cause of cancer mortality. It usually arises in cirrhotic liver, where chronic inflammation and fibrosis create a tumor-permissive microenvironment. Dysregulation of microRNAs (miRNAs), particularly upregulation of the oncomiR miR-221 and loss of the tumor suppressor miR-199a-3p represent key drivers of liver carcinogenesis. The TG221 transgenic mouse, designed to overexpress miR-221 in hepatocytes, provides a relevant in vivo platform for mechanistic studies and for testing preventive and therapeutic approaches. The TG221 model recapitulates miR-221-driven tumorigenesis, including suppression of p27, p57 and Bmf. It is characterized by steatohepatitic injury and accelerated tumor formation after genotoxic challenge. In the cirrhotic CCl_4_-induced background, TG221 mice develop fibrosis and cirrhosis followed by dysplastic and malignant lesions, mirroring the natural history of human HCC. Metformin administered during early fibrosis prevented macroscopic tumor formation and suppressed PI3K/AKT/mTOR signaling. Anti-miR-221 and miR-199a-3p mimics reduced tumor burden, restored tumor-suppressive pathways and improved liver integrity, thus indicating feasible chemopreventive strategies. From a therapeutic point of view, miR-199a-3p replacement synergized with palbociclib and overcame sorafenib resistance. A miR-199a-3p-responsive oncolytic adenovirus achieved tumor-selective replication with minimal toxicity. This review highlights the importance of the TG221 transgenic mouse as a powerful model for studying miRNA-driven hepatocarcinogenesis and enables preclinical evaluation of RNA-based chemopreventive and therapeutic approaches. Metformin, miRNA inhibition, miRNA replacement and miRNA-guided viral therapies emerge as promising approaches for advancing precision prevention and treatment strategies in HCC.

## 1. Introduction

Hepatocellular carcinoma (HCC) represents a major global health burden and is the third leading cause of cancer-related mortality worldwide [1]. Its poor prognosis largely reflects late-stage diagnosis and the limited efficacy of available systemic therapies, which typically provide only transient disease control without achieving durable remission.

HCC arises through a multistep process involving chronic liver injury, inflammation, fibrosis, cirrhosis, and ultimately malignant transformation. The majority of cases develop in the setting of chronic liver disease, commonly associated with hepatitis B or C virus infection, excessive alcohol consumption, or metabolic dysfunction leading to non-alcoholic fatty liver disease (NAFLD) and cirrhosis [2].

Cirrhosis, a preneoplastic condition present in approximately 80% of HCC patients, establishes a tumor-permissive microenvironment characterized by chronic inflammation, oxidative stress, dysregulated regenerative responses and genomic instability. Because hepatocarcinogenesis progresses through well-defined pathological stages over several years, there is a substantial opportunity to identify high-risk individuals who may benefit from targeted surveillance and preventive interventions.

Once established, HCC typically exhibits an aggressive clinical course. Despite therapeutic advances, most notably the introduction of immune checkpoint inhibitor regimens based on atezolizumab plus bevacizumab or durvalumab plus tremelimumab, median survival in patients with advanced, unresectable disease remains unsatisfactory [3]. Historically, multikinase inhibitors such as sorafenib [4] and, more recently, lenvatinib [5] constituted the mainstay of systemic therapy; however, primary and acquired resistance markedly limited their long-term benefit. They still remain important options for patients who are ineligible for or unable to tolerate immunotherapy-based treatments.

Accordingly, efforts to enhance the efficacy of existing therapies, develop effective preventive strategies and identify robust biomarkers for early detection remain critical priorities to improve patient outcomes and reduce HCC-related mortality.

A wide range of animal models is available for the study of HCC, including genetically engineered mouse models, chemically or diet-induced models (such as NASH-associated HCC), patient-derived xenografts (PDX) and tumoroids, orthotopic or transplantable models and zebrafish models (Table 1) [6,7,8,9,10,11,12]. However, these systems only rarely recapitulate the multistep progression that characterizes human HCC. Rodent models, particularly genetically engineered strains targeting oncogenic drivers, are to be combined with models that reproduce chronic liver disease and its evolution, in order to more accurately mimic the human condition. Such combined approaches can provide powerful tools for investigating HCC pathogenesis and for evaluating novel therapeutic strategies.

This review summarizes studies employing the TG221 transgenic mouse model, which has provided fundamental mechanistic insights into miRNA-driven hepatocarcinogenesis and has emerged as a versatile platform for preclinical testing of preventive and therapeutic interventions targeting molecular drivers of liver cancer. Compared with existing reviews, the present work offers a deeper and distinctive positioning of the TG221 model as a genetically defined, miR-221-driven, immunocompetent system, introducing a miRNA-centric perspective on hepatocarcinogenesis. In addition, it highlights the unique dual applicability of TG221 to both non-cirrhotic and cirrhotic HCC and underscores its direct suitability for evaluating RNA-based chemopreventive and therapeutic strategies, aspects that were not specifically addressed in previous general reviews of HCC models.

### Literature Search and Selection Approach

This article is a narrative review focused on the TG221 mouse model. The literature was identified through PubMed searches using the keywords TG221, miR-221, hepatocellular carcinoma mouse models, and miRNA therapeutics, complemented by citation tracking of seminal studies and inclusion of representative independent reviews. Potential selection bias is acknowledged, as a substantial proportion of the cited studies were authored by contributors to the present review.

## 2. The miR-221 Transgenic Mouse Model TG221

MicroRNAs (miRNAs) are short, noncoding RNAs (20–24 nucleotides) that regulate gene expression post-transcriptionally and are now recognized as key modulators of oncogenesis [30,31]. Dysregulated miRNA expression is a hallmark of HCC, where specific miRNAs critically influence tumor initiation, progression, and therapeutic resistance. Depending on their expression pattern and gene targets, miRNAs may act as oncogenes (oncomiRs) or tumor suppressors.

Among oncomiRs, miR-221 is one of the most consistently upregulated species in human HCC [32,33]. Its overexpression correlates with aggressive tumor features, including poor differentiation, vascular invasion, metastasis, and early recurrence [34,35]. Mechanistically, miR-221 promotes hepatocyte proliferation and survival by repressing key tumor suppressors, Cdkn1b/p27, Cdkn1c/p57, and the pro-apoptotic factor Bmf, as well as several other target genes, thereby driving cell-cycle progression and resistance to apoptosis in cancer cells [32,33,36,37,38,39,40,41].

**Figure 1:**
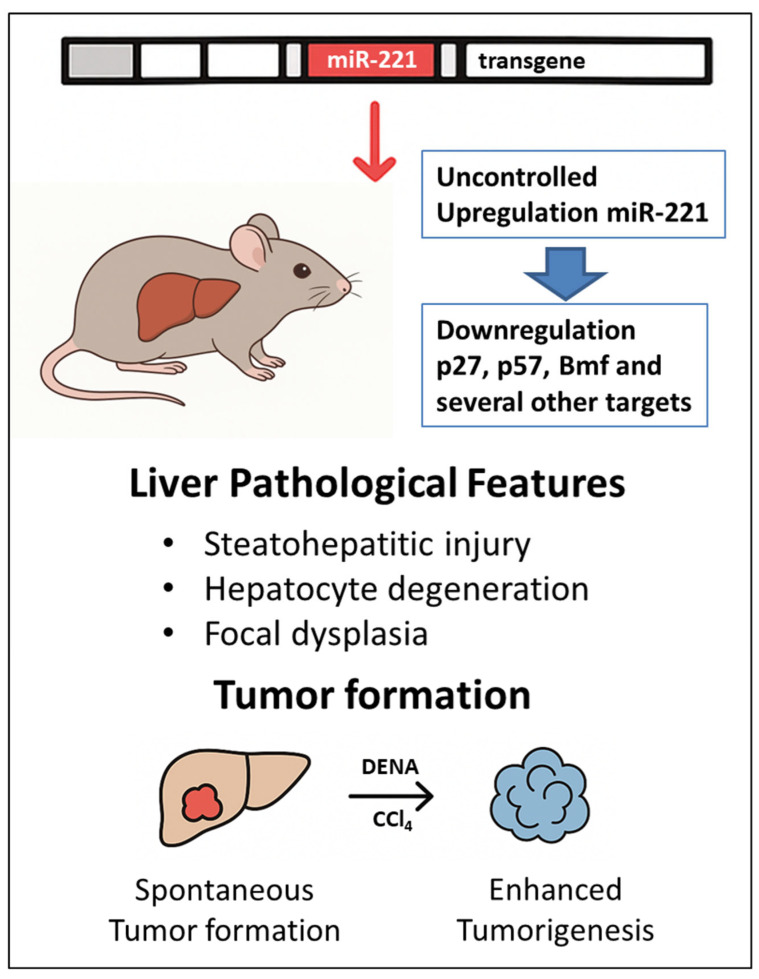
***The miR-221 transgenic mouse model (TG221) of hepatocellular carcinoma.*** Schematic overview of the generation and key molecular and pathological features of the TG221 transgenic mouse. Hepatocyte-specific expression control of miR-221 transgene results in sustained liver-restricted miR-221 upregulation. This leads to the repression of miR-221 targets, including p27, p57, and Bmf, promoting hepatocyte proliferation and survival. TG221 mice develop steatohepatitic liver injury, dysplastic lesions and spontaneous liver tumors with age, while exposure to carcinogenic challenge accelerates tumor onset and increases tumor burden. The model provides a mechanistically defined in vivo platform for studying miRNA-driven hepatocarcinogenesis. Additional details are presented in Section 2 of the text.

Given the biological and clinical relevance of miR-221 dysregulation in HCC, the development of in vivo models recapitulating its pathogenic role is essential for elucidating its function and for preclinical testing of RNA-based therapeutic and preventive strategies (Figure 1).

### 2.1. Generation and Molecular Characterization

To directly assess the oncogenic potential of miR-221 in vivo, a transgenic mouse line (TG221) was generated to constitutively overexpress miR-221 in a hepatocyte-specific manner [42]. The construct, derived from an expression vector including a chimeric regulatory cassette combining the hepatocyte-specific α1-antitrypsin promoter with the hepatitis B virus enhancer II, ensured strong liver-restricted transcription. The murine mmu-miR-221 locus was cloned downstream of this cassette, and the transgene was microinjected into fertilized B6D2F2 oocytes.

Quantitative PCR confirmed sustained hepatic overexpression of miR-221 across development and adulthood, at levels comparable to those observed in human HCC. Western blot analyses demonstrated marked downregulation of canonical miR-221 targets, including p27, p57, and Bmf, in the liver of TG221 mice relative to wild-type (WT) controls.

TG221 tumors also recapitulated miRNA signatures characteristic of human HCC, showing upregulation of miR-221 and miR-21 and downregulation of hepatocyte-specific miRNAs miR-122 and miR-199a-3p, reflecting conserved oncogenic pathways that drive hepatocarcinogenesis.

### 2.2. Pathological and Molecular Features

Liver of TG221 mice exhibits steatohepatitic injury, hepatocyte degeneration, and focal dysplasia. Approximately 50% of male TG221 mice develop spontaneous hepatic tumor nodules between 9 and 12 months of age. Although these lesions arise in non-cirrhotic livers, the accompanying metabolic alterations and steatosis recapitulate key features of human HCC.

When challenged with the carcinogen diethylnitrosamine (DEN), all male TG221 mice developed multiple liver tumors within four months, demonstrating a strong synergistic effect between miR-221 overexpression and genotoxic injury. Compared with DEN-treated WT controls, TG221 mice exhibited an earlier development and a significantly greater tumor burden, with increased nodule number and size.

In line with human HCC, the TG221 mouse model exhibits a markedly higher susceptibility to liver cancer in males. Female TG221 mice do not develop spontaneous tumors and only a fraction of females develop HCC after 12 months following DEN challenge. Although this sex bias has not been directly investigated in this model, it is plausible that, similarly to human, androgens promote hepatocarcinogenesis in males through activation of androgen receptor (AR) signaling. In contrast, estrogens in females exert a protective effect by suppressing pro-inflammatory cytokine production (e.g., interleukin-6), thereby reducing oxidative stress and fibrogenesis.

## 3. TG221 Model and HCC Prevention

Hepatocarcinogenesis is a decades-long process that unfolds through chronic inflammation, progressive fibrosis and cirrhosis. This extended latency provides a unique window of opportunities for prophylactic intervention. The TG221 model, given its pronounced predisposition to liver tumorigenesis, represents a powerful system for evaluating chemopreventive strategies.

**Figure 2:**
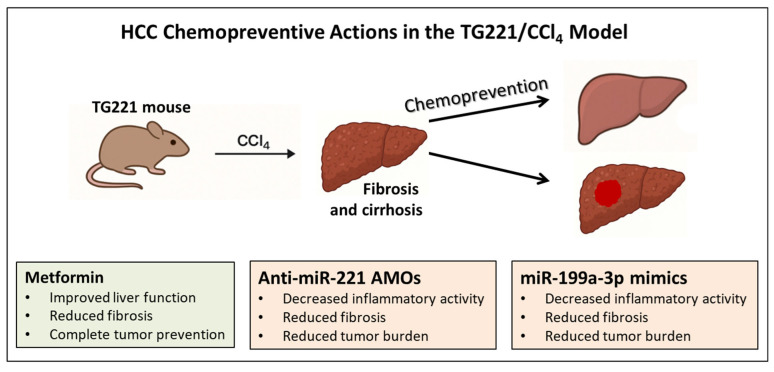
***TG221 model and HCC prevention.*** Schematic representation of the TG221 transgenic mouse model combined with chronic liver injury to investigate hepatocellular carcinoma prevention. Chronic CCl_4_ administration induces the development of progressive fibrosis, cirrhosis and tumor (represented as a red spot in cirrhotic liver) in TG221 mice, creating a tumor-permissive microenvironment that mirrors the most common milieu of human HCC. Within this context, chemopreventive strategies are evaluated, including metformin treatment, which attenuates fibrosis and prevents macroscopic tumor formation, and miRNA-based interventions, such as miR-221 inhibition and miR-199a-3p replacement, which block the progression of dysplastic lesions, reduce tumor burden and restore tumor-suppressive pathways. The figure highlights the utility of the TG221/CCl_4_ model as a platform for testing preventive approaches targeting early stages of hepatocarcinogenesis. Additional details are presented in Section 3 of the text.

A major limitation of existing HCC mouse models is that tumors arise in histologically normal livers, failing to reflect the fibrotic/cirrhotic milieu typical of human disease. Moreover, chemically induced cirrhosis in animal models (e.g., by chronic CCl_4_ administration) rarely progresses to cancer [43,44,45,46]. To overcome these limitations, cirrhosis was induced via chronic oral CCl_4_ administration in TG221 mice. Histopathology revealed extensive fibrosis, nodular regeneration and activation of fibrotic and steatosis markers. Importantly, unlike WT controls, CCl_4_-treated TG221 mice developed dysplastic and neoplastic lesions consistent with HCC, thereby providing a preclinical model that faithfully recapitulates the natural history of human HCC [47,48] (Figure 2).

### 3.1. Chemopreventive Action of Metformin

This combined CCl_4_/TG221 model was used to test chemopreventive interventions. Metformin, an antidiabetic drug epidemiologically linked to reduced HCC incidence in type 2 diabetic patients [49,50,51], was evaluated for preventive action in nondiabetic liver disease. Beginning at fibrosis onset, mice received metformin (300 mg/kg/day, orally) for 24 weeks.

Metformin improved liver function, attenuated fibrosis by suppressing stellate cell activation and collagen deposition (α-SMA, Col1a1, Col3a1, Col4a1), reduced lipid accumulation via LKB1-AMPK activation and inhibitory phosphorylation of acetyl-CoA carboxylase and, most notably, completely prevented macroscopic tumor development in CCl_4_-challenged TG221 mice. Transcriptomic profiling showed suppression of PI3K/AKT/mTOR, KRAS and mTORC1 signaling, accompanied by enhanced apoptosis [47].

These findings indicate that early administration of metformin in fibrotic, non-diabetic livers prevented progression to cirrhosis and blocked hepatocarcinogenesis by reprogramming metabolic and survival pathways, thereby providing the experimental basis for repurposing metformin as a safe, low-cost chemopreventive approach for patients with liver fibrosis, independent of their diabetic status.

The metformin dose used in TG221 mice (300 mg/kg/day) corresponds, after allometric scaling [52,53], to exposures achievable in patients with type 2 diabetes receiving standard therapeutic doses (approximately 1.5–2 g/day), supporting the translational relevance of this chemopreventive approach in metabolically at-risk or advanced fibrosis populations, supporting its consideration for HCC chemoprevention trials in at-risk selected groups.

### 3.2. Targeting miRNA Dysregulation for Chemoprevention

miRNAs serve as master regulators of hepatocarcinogenesis, suggesting that early correction of alterations in pathogenic miRNAs could represent a promising preventive approach. In the TG221/CCl_4_ model, inhibition of the oncogenic miR-221 and replacement of the tumor-suppressive miR-199a-3p were evaluated.

Systemic delivery of anti-miR-221 oligonucleotides or miR-199a-3p mimics, facilitated by the use of lipid nanoparticles as vehicles [54], effectively blocked the malignant progression of dysplastic nodules, significantly reducing tumor number and size without detectable toxicity [48]. Compared with controls, treated animals exhibited a marked reduction in both number and size of hepatic nodules, along with a shift toward a loss of dysplastic phenotype. Moreover, treated mice showed decreased inflammation, reduced fibrosis and reactivation of tumor-suppressive pathways, underscoring the central role of these miRNAs in the earliest steps of liver cell transformation [48]. Together, these results demonstrate that targeted miRNA modulation can reprogram the tumor-permissive microenvironment, leading to a stop of hepatocarcinogenesis at a precancerous stage. To this end, the TG221/CCl_4_ model provided a robust platform for preclinical evaluation of RNA-based chemopreventive strategies.

## 4. TG221 Model and miRNA-Based Therapeutic Strategies

Despite recent advances, systemic therapy for advanced HCC remains suboptimal. Sorafenib, the first agent to confer a survival benefit, is limited by toxicity and inevitable resistance. Next-generation TKIs offer modest benefits. Immunotherapy regimens improve survival in about one-third of patients.

**Figure 3:**
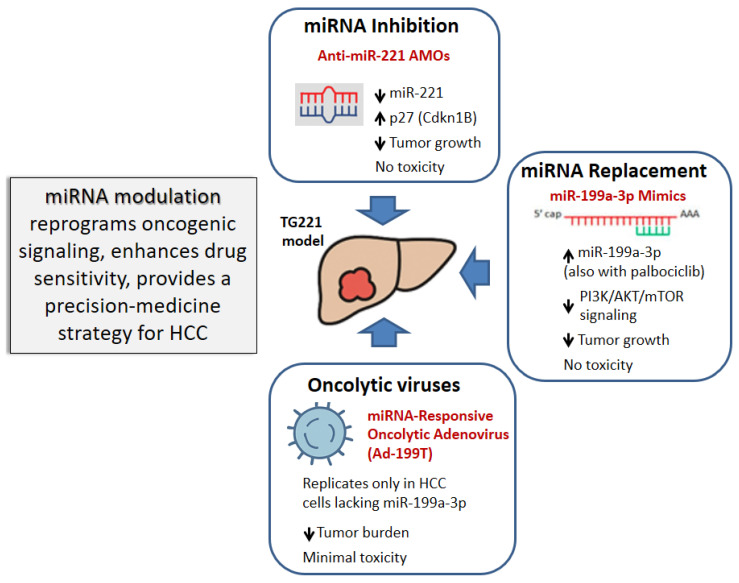
***TG221 Model and miRNA-Based Therapeutic Strategies***. Schematic overview of the therapeutic approaches evaluated in the TG221 transgenic mouse model of hepatocellular carcinoma. (4.1) miR-221 inhibition: systemic delivery of chemically modified anti-miR-221 oligonucleotides restores expression of canonical miR-221 targets (p27, p57, Bmf), leading to cell-cycle arrest and reduction in tumor number and size in TG221 mice with established HCC. (4.2) miR-199a-3p replacement: administration of miR-199a-3p mimics suppresses oncogenic signaling by targeting mTOR and PAK4, downregulates FOXM1 activity, which results in antitumor activity. (4.3) Combination strategies: miR-199a-3p replacement synergizes with the CDK4/6 inhibitor palbociclib by concurrently blocking cell-cycle progression and compensatory AKT/mTOR activation, producing enhanced tumor regression and activity in sorafenib-resistant settings. (4.4) Oncolytic virotherapy: a miR-199a-3p-regulated oncolytic adenovirus exploits differential miRNA expression between normal hepatocytes and tumor cells to achieve tumor-selective viral replication, effective tumor reduction and minimal hepatotoxicity. Together, these approaches illustrate the versatility of the TG221 model as a preclinical platform for evaluating miRNA-directed therapeutic and combinatorial strategies in HCC.

The TG221 model offers a physiologically relevant platform for evaluating miRNA-based therapeutics and combination strategies (Figure 3).

### 4.1. MiRNA Inhibition

The study by Callegari et al. [42], which led to the development of the TG221 transgenic mouse, established the role of miR-221 as a tumor driver as well as the therapeutic feasibility and safety of miR-221 silencing as an antitumor approach. Synthetic anti-miR oligonucleotides (AMOs), have proven effective for silencing miRNA activity in vivo [55,56]. In the TG221 model, intravenous delivery of synthetic 2′-O-methyl modified anti-miR-221 oligonucleotides (anti-miR-221 AMOs) successfully silenced miR-221 expression in the liver and the circulatory system, which was functionally confirmed by a concurrent increase in Cdkn1b/p27 protein expression, thus restoring a mechanism for cell cycle control. Crucially, systemic administration of AMOs to DEN-treated TG mice resulted in a significant reduction in both the number and the size of liver tumor nodules compared to untreated controls, demonstrating that miR-221 can be effectively targeted to reduce tumor burden. As reported by Krützfeldt et al., in vivo silencing of microRNAs using antagomirs is relatively long-lasting; in their seminal study, miR-122 remained undetectable for up to 23 days following a single systemic administration [56]. Consistent with these findings, in the TG221 model, mice received repeated intravenous injections of anti-miR-221 antagomirs every 15 days (10 mg/kg in saline at 60, 75, and 90 days of age). Throughout the treatment period, hepatic miR-221 levels were significantly and persistently reduced [42]. This successful in vivo inhibition, achieved without appreciable toxicity, confirms the therapeutic potential of anti-miRNA approaches for liver cancer [42].

### 4.2. MiRNA Replacement

MicroRNAs (miRNAs) have emerged as experimental therapeutic molecules against cancer, offering potential strategies such as the restoration of tumor suppressor miRNAs [30,57,58,59]. MiR-199a-3p is highly expressed in normal liver but downregulated in virtually all HCCs, a decrement correlated with poor prognosis. Given the downregulation of miR-199a-3p also in TG221 tumors, the therapeutic efficacy of a miRNA replacement strategy, based on miR-199a-3p mimic molecules, was investigated in the TG221 mouse model. MiR-199a-3p replacement was achieved using unmodified single-stranded RNA oligonucleotides, which are characterized by low intrinsic stability and whose biological effects typically fade within three days. Accordingly, in vivo administration was performed intraperitoneally at a dose of 5 mg/kg, three times per week for three consecutive weeks, using a lipid-nanoparticle-based formulation to enhance oligonucleotide stability, bioavailability and cellular uptake [60].

The administration of miR-199a-3p mimics in TG221 mice harboring liver cancer led to a significant reduction in both the number and the size of tumor nodules compared to control animals. Mechanistically, this anti-tumor activity results from the enforced expression of miR-199a-3p, which effectively downregulates in vivo its direct targets MTOR (mechanistic target of rapamycin) and PAK4 (p21 activated kinase 4), thereby suppressing PI3K/AKT/mTOR signaling This modulation ultimately led to the repression of the oncogenic transcription factor FOXM1, which is otherwise overexpressed in several solid tumors, including HCC and is associated with poor prognosis [61,62]. Remarkably, the anti-tumor activity achieved by miR-199a-3p mimics in the TG221 model was comparable to that obtained using sorafenib, in tumor-bearing TG221 mice with comparable disease stage, using matched treatment windows and tumor burden endpoints (nodule number and size). These results suggest that miR-199a-3p may be considered a promising HCC therapeutic option.

### 4.3. Combination Strategies and Sorafenib Resistance

The above findings established the basis for designing miRNA-based combination strategies. The miR-199a-3p mimics were found to act synergistically with the CDK4/6 inhibitor palbociclib. In fact, while palbociclib stops cell-cycle progression, it also triggers compensatory AKT activation, a response that miR-199a-3p can effectively suppress, thus providing the rationale for combining the two agents.

In TG221 mice bearing established liver tumors, the palbociclib plus miR-199a-3p mimics regimen produced the greatest reduction in tumor nodule volume compared with single-agent palbociclib, miR-199a-3p mimics, sorafenib, or control treatments. Importantly, body weight remained stable, indicating that the combination did not cause overt toxicity. Mechanistically, the dual regimen simultaneously inhibited the mTOR and CDK/RB1 pathways while preventing the compensatory AKT activation, resulting in enhanced antitumor activity with good tolerability [63].

Because the palbociclib–miR-199a-3p combination regimen does not include sorafenib, its activity was assessed in sorafenib-resistant cells, where it was indeed found to be effective [63]. In xenograft models generated from sorafenib-resistant HCC cells, enforced miR-199a-3p expression significantly enhanced the antitumor response to palbociclib. In a paired implantation design, AAV-miR-199a-3p transduced cells were injected into one flank, while AAV-control cells were implanted into the opposite contralateral flank, enabling direct intra-animal comparison of treatment effects. As anticipated and consistent with the resistant phenotype, sorafenib exerted minimal activity in both xenograft types. In contrast, across all animals, xenografts derived from miR-199a-3p-expressing cells, but not control-transduced cells, exhibited a markedly reduced growth in response to palbociclib. These results demonstrated that the combination of palbociclib- and miR-199a-3p-based strategies represents a potential approach to overcome sorafenib resistance in HCC [63]. Collectively, these findings highlight miR-199a-3p replacement as a potent sensitizer of CDK4/6 inhibition and support combined miRNA-targeted and cell-cycle-directed strategies as a promising therapeutic approach for HCC.

MiR-221 has also been implicated in sorafenib resistance through suppression of Caspase-3-mediated apoptosis [64], suggesting that the TG221 model could be employed for testing the use of anti-miR-221 in overcoming sorafenib resistance.

### 4.4. Oncolytic Viruses

Differential miRNA expression between normal hepatocytes and tumor cells can potentially be exploited to enhance the selectivity of oncolytic virotherapy. Oncolytic viruses are genetically engineered viruses that are designed to selectively infect, replicate within and kill cancer cells while sparing the normal ones. They represent an emerging class of cancer therapeutics that leverage both direct tumor cell lysis and stimulation of anti-tumor immunity. Ongoing research and clinical trials suggest that oncolytic viruses will play an increasingly important role in multimodal cancer treatment strategies. Multiple clinical trials are ongoing in solid tumors, often in combination with checkpoint inhibitors or radiation therapy [65,66,67,68]. The first FDA-approved oncolytic virus therapy was Talimogene laherparepvec (T-VEC), an HSV-1-based oncolytic virus engineered to express GM-CSF approved for metastatic melanoma [69].

To enhance tumor selectivity, a miRNA-responsive oncolytic adenovirus (Ad-199T) was designed to exploit the differential expression of miR-199a-3p, abundant in normal liver but absent in HCC. In TG221 mice, intravenous administration of Ad-199T achieved preferential replication within tumors, reducing tumor burden by 2–3-fold compared to controls and exhibiting minimal hepatotoxicity. These results highlight the potential of miRNA-regulated oncolytic viral therapy as a precision strategy for HCC [70].

Nevertheless, the approach presents some relevant translational constraints, including reduced viral replication in cirrhotic tissue, innate immune activation and anti-adenovirus immunity, which makes the approach as a proof-of-concept strategy requiring further optimization.

## 5. Conclusions

The TG221 transgenic mouse model has been proven essential for elucidating the oncogenic role of miR-221 in hepatocarcinogenesis. Its miRNA expression profile closely mirrors human HCC, including upregulation of oncomiRs (miR-221 and miR-21) and downregulation of tumor suppressors miR-122 and miR-199a-3p. Beyond mechanistic value, TG221 is highly versatile for translational research, enabling evaluation of miRNA inhibition, miRNA replacement, oncolytic strategies, and drug-combination regimens.

The incorporation of a cirrhotic background (CCl_4_/TG221) further enhances translational relevance, providing a unique platform for testing chemopreventive interventions in high-risk liver disease.

Compared with the preclinical HCC models summarized in a recent published review [71], the TG221 transgenic mouse offers a distinct and complementary way to study liver cancer. Table 2 highlights how TG221 differs from commonly used mouse models, and Table 3 illustrates the specific gaps this model helps to address.

First, unlike chemically or diet-induced models (such as DEN, CCl_4_, Western diet, or NASH-like regimens), which depend on random DNA damage and long-term inflammation to create genetically diverse tumors, TG221 provides a genetically defined system. It is driven by overexpression of miR-221, one of the most consistently upregulated oncomiRs in human HCC. This leads to tumors that are more controlled, reproducible, and easier to interpret mechanistically.

Second, while xenograft and patient-derived xenograft (PDX) models reproduce human tumor architecture, they require immunodeficient mice, making it impossible to study interactions between the tumor and the immune system. In contrast, TG221 mice develop tumors in a fully immunocompetent host, and the model can be paired with cirrhosis-inducing treatments to better mimic the inflammatory environment of advanced liver disease.

Third, compared with traditional genetically engineered mouse models based on activating single oncogenes or deleting tumor suppressors (such as Myc, β-catenin, or PTEN), TG221 uniquely combines metabolic injury with miRNA dysregulation. As a result, the mice develop steatohepatitic features and a miRNA expression profile that closely mirrors human HCC.

Finally, although hydrodynamic transposon models (SB/HTVI) can quickly generate tumors driven by defined sets of oncogenes, they generally lack the chronic liver damage seen in human disease. TG221, however, can model both spontaneous and injury-accelerated tumor development. Exposure to DEN or CCl_4_ produces strong synergy, leading to highly penetrant and faster-forming tumors.

Taken together, these characteristics position TG221 as a valuable middle ground between classical GEMs and highly variable chemical or dietary models, biologically relevant, miRNA-driven, immunocompetent, and metabolically altered system with strong translational potential.

This model also presents some important limitations [72,73]. In particular, TG221 tumorigenesis is driven by a single dominant oncomiR (miR-221), which may not fully capture the genetic complexity and multi-driver evolution that characterize human HCC. Moreover, although microRNA sequences are highly conserved across species, their basal expression levels, effective target repertoires and regulatory networks may differ between species (https://www.mirbase.org/) [74,75,76]. In addition, there are relevant species-specific differences in liver metabolism and immune responses that must be considered when translating findings from TG221 mice into clinical settings. Species-dependent variations in hepatic metabolism, drug clearance and immune regulation can significantly influence pharmacokinetics and the immune-mediated effects, in human hepatocarcinogenesis. For example, mice and humans express largely overlapping cytochrome P450 (CYP) enzyme families; however, the relative abundance of individual isoenzymes differs markedly between species. In addition, key enzymes such as CYP2E1 display distinct kinetic properties and regulatory mechanisms, while several isoforms (e.g., Cyp3a11, Cyp3a13) are mouse-specific. Consistent with these differences, mice exhibit faster drug metabolism and higher hepatic clearance, resulting in shorter drug half-lives. This explains why higher mg/kg doses are often required in murine models to achieve systemic exposures comparable to those observed in humans. Furthermore, the murine liver is more prone to rapid steatosis development, reflecting species-specific differences in lipid and glucose metabolism [77,78,79]. From an immunological perspective, mice display notable species-specific differences in hepatic immune responses, including higher basal activation of Kupffer cells and distinct expression patterns and sensitivities of Toll-like receptors (TLRs). These differences contribute to hepatic inflammation and tumor microenvironments that are not fully overlapping between mice and humans. Differences also extend to adaptive immunity, with substantial variation in T helper cell subsets (Th1/Th2/Th17) and in the expression and regulation of immune checkpoint molecules such as PD-1 and CTLA-4. Collectively, these factors can result in divergent responses to immuno-oncological therapies and underscore the limited predictive power of murine models for immunotherapy in HCC [80,81]. Recognizing these limitations may help to better contextualize the appropriate applications of the model while preserving its strengths.

Beyond their mechanistic role, miR-221 upregulation and miR-199a-3p loss represent clinically relevant expression patterns that may be leveraged as biomarkers for HCC risk stratification and patient selection. Meta-analytic and cohort-level evidence supports an association between elevated miR-221 and adverse prognosis [64,82], while reduced miR-199a-3p is repeatedly observed in HCC and aligns with tumor-suppressive pathway disruption. In the liquid biopsy setting, circulating miRNA panels that include miR-221 show moderate diagnostic value [83,84]. Finally, because these miRNAs map onto actionable signaling programs, miR-221 and/or miR-199a-3p profiling could support biomarker-guided trial enrichment for miRNA-targeted or rational combinations therapeutics [60,85]. Insights from TG221 studies could guide the identification of patient subgroups most likely to benefit from miRNA-targeted preventive or therapeutic strategies. Future work should further focus on preventive strategies in cirrhotic TG221 models and on combinatorial regimens targeting resistance pathways. Advances in delivery systems, including AAV vectors, innovative nanotechnologies approaches and polymeric carriers, will be critical for effective, liver-targeted modulation of pathogenic miRNA circuits.

Overall, TG221 represents a significant model for the preclinical study of miRNA-driven hepatocarcinogenesis and provides a robust bridge between molecular discovery and clinical translation.

## Figures and Tables

**Table 1:** Representative *in vivo* models for HCC investigation.

Model Category	Model (Type)	Experimental Details	References
Genetically engineered mouse models	Tet-regulated MYC-driven HCC GEMM	LAP-tTA; TRE-MYC transgenic mice develop aggressive HCC upon MYC overexpression; doxycycline turns MYC off, causing regression and differentiation of tumors.	[13]
	CRISPR/Cas9 liver TSG-KO models (Pten, Trp53, Ctnnb1)	Hydrodynamic tail-vein injection of plasmids expressing Cas9 and sgRNAs into wild-type mouse liver → in vivo editing of Pten, Trp53 alone or combined, producing autochthonous liver tumors.	[14]
	Sleeping Beauty hydrodynamic transfection models	Sleeping Beauty transposon + hydrodynamic injection to overexpress oncogenes (e.g., MYC, NRAS^G12V, AKT, β-catenin) and/or shRNA knockdown of TSGs in hepatocytes → rapid, genotype-defined HCC.	[15]
	HDI model with Pten/p53 KO + c-Met + Δ90-β-catenin	Hydrodynamic co-delivery of plasmids to knock out Pten/Trp53 and overexpress c-Met and Δ90-β-catenin; luciferase tag for non-invasive bioluminescence imaging of tumor burden.	[16]
Chemically/ diet-induced models	Classic DEN-based chemical HCC	Single i.p. diethylnitrosamine (DEN) injection in young mice → chronic genotoxic injury and inflammation → HCC over ~8–10 months; often combined with CCl_4_ or partial hepatectomy.	[17]
	High-fat diet (HFD) + low-dose CCl_4_ NASH–HCC model	C57BL/6J mice on high-fat, high-fructose, high-cholesterol diet plus weekly low-dose CCl_4_ for 12–24 wks → steatosis → steatohepatitis → fibrosis (12 wks) and HCC (24 wks).	[18]
	High-fat-diet-conditioned orthotopic HCC	Mice pre-fed high-fat diet to induce steatosis/steatohepatitis; then syngeneic murine HCC cells orthotopically implanted into the liver; model captures HCC in a lipotoxic, pro-tumorigenic microenvironment.	[19]
Orthotopic models	Orthotopic HCC in CCl_4_-induced cirrhotic liver	12 wks CCl_4_ to induce micronodular cirrhosis; then surgical intrahepatic implantation of syngeneic HCC cells (e.g., Hepa1-6) under microscopy; recapitulates HCC arising in cirrhotic, inflamed, fibrotic liver.	[20]
	HFD-conditioned syngeneic orthotopic HCC model	High-fat-diet preconditioning, then implantation of syngeneic HCC fragments/cells into liver; supports studies of diet-induced microenvironment changes on tumor growth and drug response.	[19]
	Immune-competent orthotopic Hep-55.1c vs. iAST models	Orthotopic models: transplanted Hep-55.1c tumors and transgenic iAST tumors in immune-competent mice; characterized for morphology, immune infiltrates, mutational burden and response to PD-1/CTLA-4 blockade.	[21]
Patient-derived xenografts (PDX)	Human HCC PDX in immunodeficient/humanized mice (overview)	Review summarizing engraftment of fresh HCC/ICC tumor fragments or cell suspensions into NSG/NRG or humanized mice; some models incorporate patient-matched stroma or immune components.	[22]
	Patient-derived models incl. PDX, PDO, PCTS, PDTS	Comprehensive review of HCC/iCCA patient-derived xenografts, organoids, precision-cut tissue slices, spheroids and iPSC-based systems, with emphasis on tumor–matrix and tumor–immune interactions.	[23]
	Primary liver-cancer-derived tumoroids (HCC/CC/CHC organoids)	Organoids (“tumoroids”) grown from resected human HCC, cholangiocarcinoma and combined HCC/CC tumors in Matrigel with defined growth factors; long-term expansion; xenografting recapitulates donor tumor histology and metastasis.	[24]
	Patient-derived liver cancer cell models and PDOs (precision medicine focus)	Review of recent liver cancer PDO and cell-line generation → large panels of genomically annotated HCC models for pharmacogenomic and “avatar”-style drug testing.	[25]
	Organotypic precision-cut tissue slices from primary liver cancer	Ex vivo culture of precision-cut tissue slices from HCC/iCCA/mixed tumors maintaining native architecture, vasculature and immune cells; used for short-term drug testing.	[26]
Zebrafish HCC models	Overview of zebrafish HCC models (transgenic, chemical, xenograft)	Review of liver-specific oncogene transgenic lines, chemical carcinogenesis and xenograft models in zebrafish larvae; highlights imaging.	[27]
	Transgenic zebrafish for modeling HCC	Liver-specific, inducible expression (e.g., using fabp10 or liver fatty acid binding protein promoters) of human or fish oncogenes (KRAS G12V, Myc, CTNNB1 variants); tumors arise in weeks.	[28]
	Inducible liver-cancer zebrafish models (multi-oncogene panel)	Describes LexPR/Gal4-UAS-type inducible liver-specific expression of various oncogenes and combinations in zebrafish; allows temporal control of tumor initiation and regression.	[29]

**Table 2:** Comparative analysis of the TG221 transgenic model versus major HCC model categories.

Model Category	Key Features of Model	Advantages of TG221	Limitations of TG221
Chemically and Dietary Induced Models (DEN, CCl_4_, WD/HFD, NASH models)	Induce HCC via genotoxic injury, inflammation, fibrosis; high heterogeneity; long latency; strain-dependent; resemble chronic human liver disease.	• Genetically defined, mechanistically interpretable oncogenic driver (miR-221).	• Lack of intrinsic fibrosis/inflammation unless CCl_4_ is added.
		• Spontaneous steatohepatitic changes without dietary manipulation.	• Lower genetic heterogeneity.
		• Accelerated tumorigenesis with DEN, reducing inter-animal variability.	• Longer spontaneous tumor latency than DEN-based models.
		• Operates in immunocompetent background.	
Xenograft Models	Rapid, reproducible tumor formation; human tumor tissue; high translational value; require immunodeficient hosts; lack microenvironmental fidelity in heterotopic sites.	• Fully immunocompetent setting enables immune–tumor interaction studies.	• Xenografts better reproduce human tumor genomics and architecture.
		• Tumors arise within native mouse liver microenvironment.	• TG221 provides only mouse-derived tumors and cannot assess human-specific drivers.
		• Compatible with cirrhosis induction (CCl_4_/TG221).	
Patient-Derived Xenografts (PDX)	Preserve patient-specific heterogeneity and stromal interactions; valuable for personalized therapy testing; limited engraftment; require immunodeficiency.	• Immunocompetent and genetically tractable.	• Lack the human tumor heterogeneity and fidelity of PDX.
		• Ideal for evaluating miRNA-directed therapies (antagomirs, replacement).	• Cannot model patient-specific mutational signatures.
		• More cost-effective and reproducible than PDX.	
Genetically Engineered Mouse Models (GEM) (Myc, TGF-α, β-catenin, PTEN KO, HBx, etc.)	Precise genetic alterations; immunocompetent; stable inheritance; often lack metabolic features; may have long latency or embryonic lethality.	• miRNA-driven oncogenesis closely mirrors human HCC miRNA signatures (↑ miR-221/21, ↓ miR-122/199a).	• Driven by a single oncomiR → limited modeling of multi-driver tumor evolution.
		• Reproduces metabolic injury and steatohepatitis absent in many GEMs.	• Spontaneous tumor onset slower than some high-penetrance GEMs (e.g., Myc).
		• Compatible with fibrosis induction to model cirrhosis-associated HCC.	
Hydrodynamic Transposon Models (SB/HTVI)	Rapid, multi-driver, customizable tumor induction; recapitulate specific molecular subclasses; target hepatocytes; lack chronic liver disease background.	• Models chronic injury-accelerated HCC when combined with CCl_4_.	• Cannot replicate the speed and multi-hit flexibility of SB/HTVI.
		• Provides spontaneous and carcinogen-accelerated tumorigenesis in an immunocompetent, metabolically altered liver.	• Less suited to subtype-specific modeling (e.g., MET/β-catenin, AKT/Ras).
		• More physiologically relevant metabolically than HTVI tumors.	

**Table 3:** Strengths of the TG221 transgenic mouse model in the context of preclinical HCC research.

Strength	Description and Scientific Relevance
miRNA-Driven Oncogenesis (miR-221 Overexpression)	TG221 uniquely models HCC initiated by deregulated miRNA expression—specifically miR-221, one of the most consistently upregulated oncomiRs in human HCC. No other GEM or chemical model in the review reproduces a miRNA-centric driver lesion.
Human-Like miRNA Signature	TG221 recapitulates the miRNA landscape of human HCC, including ↑ miR-221/miR-21 and ↓ miR-122/miR-199a-3p. This mirrors conserved oncogenic pathways not replicated in other HCC models.
Integration of Metabolic Injury and Oncogenesis	Spontaneously develops steatohepatitic changes and metabolic alterations without the need for dietary induction, reflecting key features of metabolic-associated HCC.
Dual-Context Tumorigenesis (Non-Cirrhotic and Cirrhotic Backgrounds)	Can generate spontaneous tumors in healthy livers and HCC in cirrhotic livers when combined with CCl_4_. This dual capability is rare among GEM models and not achievable in xenograft/PDX systems.
Strong Synergy with DEN	When exposed to DEN, TG221 yields rapid, fully penetrant, and high-burden HCC, enabling accelerated carcinogenesis studies with reduced variability.
Preserved Immunocompetence	Maintains intact immune–tumor interactions, unlike xenograft/PDX systems. Enables evaluation of immunomodulatory therapies in a miRNA-driven HCC context.
Direct Suitability for miRNA-Targeted Therapies	Ideal platform for testing antagomirs, miRNA mimics, combinational regimens, viral vectors, and novel molecular inhibitors targeting miRNA-regulated pathways, applications not feasible in most models described in the review.
Mechanistic Clarity with Translational Relevance	The single, well-defined driver lesion allows precise mechanistic dissection of pathways downstream of miR-221 while still reflecting clinically relevant metabolic and molecular alterations.
Stable and Reproducible Tumor Onset	TG221 offers predictable disease progression, unlike dietary or toxin-based models which display high inter-animal variability due to stochastic mutagenesis and variable fibrosis progression.

## Data Availability

No new data were created or analyzed in this study. Data sharing is not applicable to this article.
